# Does anatomic single-bundle ACL reconstruction using hamstring autograft produce anterolateral meniscal root tearing?

**DOI:** 10.1186/s40634-017-0093-5

**Published:** 2017-05-22

**Authors:** Sebastián Irarrázaval, Angel Masferrer-Pino, Maximiliano Ibañez, Tamer M. A. Shehata, María Naharro, Joan C. Monllau

**Affiliations:** 1 Department of Orthopaedic Surgery, School of Medicine, Pontificia Universidad Católica de Chile, Diagonal Paraguay 362, Santiago, Chile; 2 ICATME, Hospital Universitari Dexeus, Barcelona, Spain; 3 Tadawi General Hospital, Dammam, Saudi Arabia; 4 Complejo Hospitalario Universitario de Pontevedra, Galicia, Spain; 5 Department of Orthopaedic Surgery and Traumatology, Hospital del Mar, Universitat Autònoma de Barcelona, Barcelona, Spain

**Keywords:** Meniscal roots, Anterior cruciate ligament, Anterior cruciate ligament reconstruction, Lateral meniscus

## Abstract

**Background:**

To determine if tibial tunnel reaming during anatomic single-bundle anterior cruciate ligament (ACL) reconstruction using hamstring autograft can result in anterolateral meniscal root injury, as diagnosed by magnetic resonance imaging (MRI).

**Methods:**

A case series of 104 primary anatomic single-bundle ACL reconstructions using hamstring autograft was retrospectively reviewed. Pre- and post-operative (>1 year) MRIs were radiologically evaluated for each patient, with a lateral meniscus extrusion > 3 mm at the level of the medial collateral ligament midportion on a coronal MRI, to establish anterolateral meniscal root injury.

**Results:**

No patients presented radiological findings of anterolateral meniscal root injury in this case series.

**Conclusions:**

Examining a single-bundle ACL reconstruction technique using hamstring autograft that considered tibial tunnel positioning in the center of the tibial footprint, this case series found no evidence of anterolateral meniscal root injury in patient MRIs, even more than 1-year post-operation.

## Background

The menisci are essential anatomical structures for tibiofemoral congruity, stabilization, shock absorption, and, possibly, proprioception (Fithian et al. 1990; Koenig et al. 2009). Axial load dissipation is mechanically dependent on meniscal structural integrity (Kim et al. 2012; Kopf et al. 2011), and injuries involving meniscal root detachment affect root biomechanics and can lead to degenerative changes within the knee joint (Kopf et al. 2011; Allaire et al. 2008; Sung et al. 2013; Shelbourne et al. 2011; Shybut et al. 2015; Ziegler et al. 2011). Some reports have demonstrated increased levels of meniscal extrusion after anteromedial horn meniscal tears, which correlate with significant cartilage degeneration (Costa et al. 2004; Lerer et al. 2004; Mariani et al. 2015). Others have even reported that a posterior meniscal root tear has biomechanical consequences similar to total meniscectomy (Allaire et al. 2008; Shelbourne et al. 2011; Ellman et al. 2014).

Anatomic anterior cruciate ligament (ACL) reconstruction advocates for the restoration of native ligament insertion sites by using the center of the ACL footprint as a reference for tunnel positioning (Middleton et al. 2014; Fu et al. 2015). Anatomic ACL reconstruction can yield better clinical and biomechanical results than non-anatomic reconstruction (Hussein et al. 2012). However some studies have suggested that placing the tibial tunnel at the center of the tibial footprint may damage the anterolateral meniscal root (ALMR) (Laprade et al. 2014a; Laprade et al. 2014b). A cadaveric study showed that a tunnel reamed in the center of the tibial footprint can cause a significant decrease in the attachment area and, ultimately, in the strength of the ALMR (Bhatia et al. 2014). A recent study has also shown that a posterolateral location of the tibial tunnel would provide an ALMR injury (Ting & Della Valle 2017).

The purpose of this study was to determine if tibial tunnel reaming during anatomic ACL reconstruction could result in an ALMR tear, as diagnosed by magnetic resonance imaging (MRI). We hypothesized that tibial tunnel reaming during ACL reconstruction would not result in ALMR disruption.

## Methods

A series of primary anatomic ACL reconstructions performed between 2008 and 2015 by one senior surgeon were retrospectively reviewed. The inclusion criteria were 1) anatomic single-bundle ACL reconstruction using hamstring autograft, without concomitant anterolateral or posterolateral meniscal root tears, as diagnosed before or during surgery; 2) at least 1 year of follow-up control MRI; and 3) a preoperative MRI. Patients with lateral meniscal tears, multiligamentous injuries or a history of ipsilateral knee surgeries were excluded.

This study was approved by the Ethics Committee at the Department of Orthopaedic Surgery and Traumatology, Hospital del Mar, Universitat Autònoma de Barcelona (2016/7004).

### Surgical technique

**Fig. 1:**
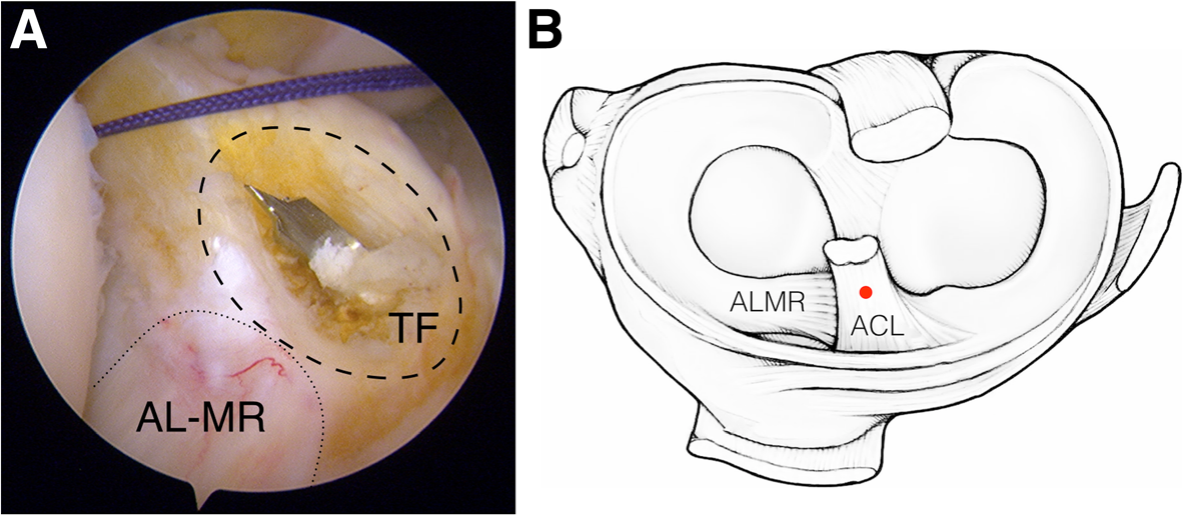
Tibial footprint center. Prior to tunnel drilling, the tibial insertion site and its peripheral structures, including the anterior root of the lateral meniscus (ALMR), were carefully examined to identify the center of the tibial footprint (TF). This point was regularly 2 to 3 mm anteromedial to the posterior margin of the ALMR (**a**: arthroscopic view; **b**: schematic view)

Classic portals for anatomic ACL reconstruction using hamstring autograft were used (i.e. anterolateral, anteromedial, and accessory anteromedial). To assist with tunnel positioning, 1 to 2 mm of the ACL stump was conserved. Femoral tunnel placement was determined at an equidistant point between the anteromedial and posterolateral femoral footprints using the BullsEye femoral guide (ConMed Linvatec, Largo, FL, USA). Tibial tunnel placement was determined using an ACL aiming guide, which was positioned at the calculated center of the ACL footprint through examination of the tibial insertion site and surrounding structures (Ziegler et al. 2011). This point was regularly 2 to 3 mm anteromedial to the posterior margin of the ALMR, which was always evaluated before reaming (Ziegler et al. 2011) (Fig. 1). A 2.4 mm guide pin was positioned using a 55° drill-guide angle. Tunnel size was established as a diameter 1 mm less than the graft diameter. After reaming, a dilator with a diameter equal to the graft diameter was inserted to smooth and compact the tunnel. The final tunnel diameter was 8 mm at minimum and 11 mm at maximum. For graft fixation, absorbable interference screws were used on the tibial side, and cortical suspension device were used on the femoral side.

### Clinical assessments

Clinical patient assessments were performed prior to surgery and periodically thereafter, starting from 1 week to at least 1 year after surgery. Meniscal symptoms were evaluated as part of the standard knee exam, including load-dependent pain, lateral joint line tenderness, posterior knee pain at full flexion, and a positive McMurray test (Ahn et al. 2010; Habata et al. 2004; Vyas & Harner 2012).

### Radiological assessments

**Fig. 2:**
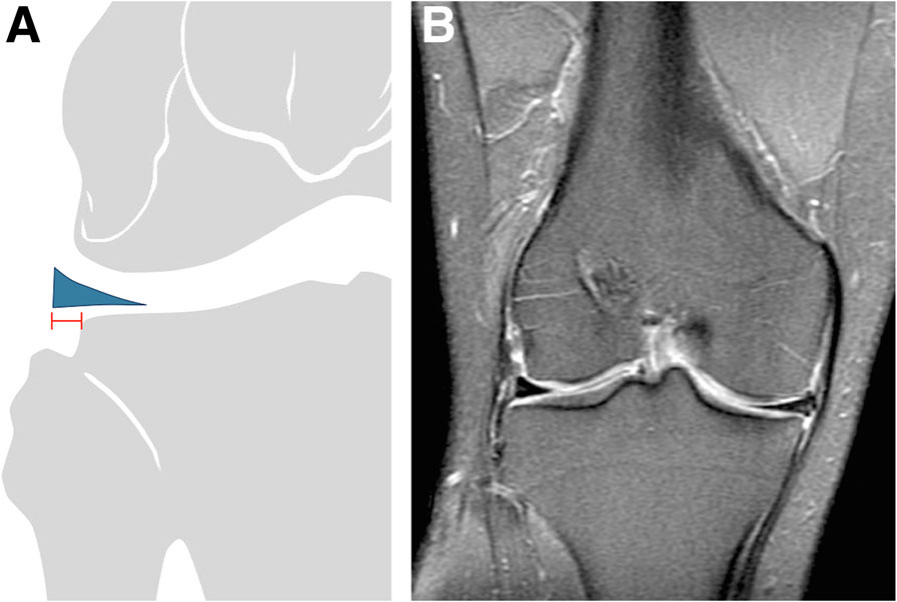
Meniscal extrusion evaluated in a coronal view MRI, at the level of the medial collateral ligament midportion. **a**) Schematic image showing the measurement of the meniscal extrusion. ≥ 3 mm is considered pathological. **b**) Coronal T2-weighted MRI showing absence of meniscal extrusion

Radiological assessments included an MRI before and at least 1 year after surgery (control exam). All postoperative MRIs were reviewed by two trained orthopedic surgeons to determine the presence of ALMR detachment. The radiological criteria was a meniscal extrusion > 3 mm at the level of the midportion of the medial collateral ligament on a coronal MRI, using at least 3 concomitant coronal slices (Bhatia et al. 2014) (Fig. 2).

### Statistical analysis

Categorical variables were presented as frequencies and percentages. The mean and range were calculated for each continuous variable. The Chi-square test was used to compare the results of groups with and without meniscal root tears. Statistical analyses were performed using SPSS 19 (SPSS Inc., Chicago, IL, USA). Significance was set at P < 0.05.

## Results

The case series included 31 females (29.8%) and 73 males (70.2%), with a mean age of 32.6 ± 14.2 years-old. The mean follow-up time was 14.6 months (range of 12–18.5 months). Isolated ACL reconstruction was performed in 85 cases (81.7%), while an associated arthroscopic procedure was performed in 19 cases (18.3%, partial medial meniscectomy). Tibial tunnel reamed diameter was 9.32 ± 0.82 mm (8 mm: 14.4%; 9 mm: 46,2%; 10 mm: 31,7%; 11 mm: 7,7%).

### Clinical findings

No patients in the evaluated series presented clinical findings of meniscal tearing 1 year after reconstruction surgery.

### Radiological findings

The average diameter of MRI-measured tibial tunnels was 9.23 ± 1.1 mm. Considering the radiological MRI criteria needed to establish ALMR detachment, no patients in the present case series presented ALMR tearing, even more than 1 year post-surgery. The medial meniscus roots were also reviewed, and no cases of anterior meniscal root tearing were found.

## Discussion

The most important finding of this case series study was that anatomic single-bundle ACL reconstruction using hamstring autograft was not related to MRI criteria of ALMR tearing, even 1 year post-operation. The assessed series was comprised of cases from a single surgeon that performed ACL reconstruction using hamstring autograft by reaming a tibial tunnel in the center of the tibial footprint.

It was recently shown that the anteromedial meniscal root can suffer iatrogenic injury during tibial tunnel creation for ACL reconstruction (Laprade et al. 2014a). This anteromedial injury might obfuscate the recognition of ALMR injury. Nevertheless, a number of cadaveric studies have indicated that anatomic single-bundle ACL reconstruction can significantly decrease ALMR attachment area and strength, as the native ACL tibial insertion is overlapped with the ALMR (Laprade et al. 2014b; Watson et al. 2015). Regarding the attachment area, some studies report that the ALMR is smaller than the anteromedial meniscal root (44.5 mm^2^ vs 93 mm^2^) (Johnson et al. 1995; Kohn & Moreno 1995). In contrast, recent anatomical and biomechanical studies provide evidence that the ALMR attachment area is 140.7 mm^2^ and overlaps up to 40.7% with the tibial ACL footprint (Ellman et al. 2014; Laprade et al. 2014b; Watson et al. 2015; Laprade et al. 2014c; Zantop et al. 2008). A recent study using scanning electron microscopy has shown that the mean percentage of ACL fibers overlapping the ALMR insertion, in the coronal and sagittal planes, was 41.0, 6 8.9 and 53.9, 6 4.3%, respectively (Steineman et al. 2017). Another study has shown that a posterolateral location of the tibial tunnel aperture within the footprint of the native ACL increases extrusion of the lateral meniscus post-reconstruction, where extrusion provides a proxy measure of injury to the anterior root (Ting & Della Valle 2017).

The relation between the ALMR and other anatomical structures has also been qualitatively and quantitatively described. Zantop et al. (Zantop et al. 2008) reported that the ALMR center was anteromedial to the apex of the lateral tibial eminence, anteromedial to the closest edge of the articular cartilage of the lateral tibial plateau, anterolateral to the center of the ACL tibial attachment, and anterior to the nearest edge of the posterior lateral meniscal root. Quantitatively, Zantop et al. (Zantop et al. 2008) further reported that the anteromedial bundle of the ACL is 5.2 mm medial and 2.7 mm posterior to the ALMR center, whereas the posterolateral bundle is 11.2 mm posterior and 4.1 mm medial to the ALMR center. Similarly, Ziegler et al. (Ziegler et al. 2011) found that the ACL center was 7.5 mm medial to the ALMR center. Ziegler et al. (Ziegler et al. 2011) also individually described the ACL bundle attachments, with the anteromedial center 8.3 mm medial to the anterior-most fibers of the ALMR, and the posterolateral center 6.6 mm medial to the posterior-most fibers of the ALMR. Finally, Luites et al. (Luites et al. 2007) observed the footprint center and ACL bundles in relation to the tibial eminences (i.e. medial and lateral intercondylar eminences), noting that the average center of the tibial footprint as a whole is approximately two-fifths the interspinous distance medially to laterally.

Nevertheless, these anatomical studies should be interpreted with caution, particularly as a systematic review by Hussein et al. (Hwang et al. 2012) found heterogeneity among the anatomic landmarks used as references for the ACL tibial footprint and in whether the footprint center was observed as a whole or according to the individual anteromedial and posterolateral bundles. Indeed, this review observed that the most consistent arthroscopic landmark for the tibial footprint was the anterior border of the posterior cruciate ligament, with the tibial footprint center 15 mm anterior to the border (Cuomo et al. 2006; Edwards et al. 2007; Heming et al. 2007). However, it is worth noting that Hussein et al. (Hwang et al. 2012) performed a systematic review of subjective interpretations and failed to provide a definitive, quantitative synthesis through meta-analyses. Considering the heterogeneity in anatomic landmarks, placement of the tibial tunnel using an existing footprint remnant might be a better approach than using bony or meniscal landmarks to restore the anatomic position of the ACL. Supporting this, a recent study showed that ACL reconstructions using an existing footprint remnant for tunnel placement provide better objective and subjective clinical results than reconstructions using bony landmarks (Lu et al. 2015).

Currently, MRIs are the best diagnostic tool for detecting ALMR tears, particularly in the absence of highly specific patient history and/or findings through physical examination (Bhatia et al. 2014). While accurate diagnosis with an MRI is dependent on image quality and the skill of the evaluator, the detection of meniscal root tears is significantly improved by using a variety of magnetic resonance sequences and interpretation signs suggestive of root tears (Choi et al. 2012; De Smet et al. 2009; Muhle et al. 2013). For these parameters, T2-weighted sequences provide the highest specificity and sensitivity values (Lee et al. 2008).

The relatively small size of meniscus roots complicates visualizing a clear tear. Therefore, examinations frequently search for a meniscal extrusion, a pathology highly correlated with root tears (Choi et al. 2010; Magee 2008). A meniscal extrusion is defined as partial or total displacement of the meniscus from the tibial articular cartilage (Lerer et al. 2004). A 3 mm extrusion on midcoronal imaging is significantly associated with articular cartilage degeneration, meniscal degeneration, complex tear patterns, and tears involving the meniscus root (Costa et al. 2004; Lerer et al. 2004). In this study, meniscal extrusion was used to assess ALMR disruption.

Worth noting, this study is limited due to its retrospective nature and since MRIs were obtained with patients in a supine position, where meniscal extrusion is theoretically worse under weight-bearing conditions. Also, anterior and posterior extrusions were not evaluated, but, to our knowledge, no method exists for evaluating these extrusions via MRI.

Future studies should aim to establish a methodology for diagnosing potential ALMR injuries associated with ACL reconstruction.

## Conclusion

Given the close anatomical relation between the ALMR and the ACL tibial footprint, it is possible that the ALMR could incur injury as a result of tibial tunnel reaming during ACL reconstruction. To assess this possibility, the current study examined a case series of single-bundle ACL reconstructions, using hamstring autograft with an average of 9.32 mm tibial tunnel reamed diameter, that considered tunnel positioning in the center of the tibial footprint. No MRI evidence for ALMR injury was found, even more than 1 year post-operation.

## Abbreviations

ACL: Anterior cruciate ligament
ALMR: Anterolateral meniscal root
MRI: Magnetic resonance imaging
